# From Expectations to Experiences: Consumer Autonomy and Choice in Personal Genomic Testing

**DOI:** 10.1080/23294515.2019.1701583

**Published:** 2019-12-30

**Authors:** Jacqueline Savard, Chriselle Hickerton, Sylvia A. Metcalfe, Clara Gaff, Anna Middleton, Ainsley J. Newson

**Affiliations:** a Faculty of Medicine and Health, Sydney School of Public Health, Sydney Health Ethics, The University of Sydney, Sydney, NSW, Australia;; b School of Medicine, Faculty of Health, Deakin University, Victoria, Australia;; c Genetics Education and Health Research, Murdoch Children’s Research Institute, Victoria, Australia;; d Department of Paediatrics, University of Melbourne, Melbourne, Victoria, Australia;; e Walter and Eliza Hall Institute of Medical Research, Victoria, Australia;; f Society and Ethics Research, Wellcome Genome Campus Society and Ethics Research Group, Hinxton, Cambridge, United Kingdom;; g Faculty of Education, University of Cambridge, Cambridge, United Kingdom

**Keywords:** personal genomics, personal autonomy, direct-to-consumer testing, genetic testing, qualitative research, bioethics

## Abstract

**Background**: Personal genomic testing (PGT) offers individuals genetic information about relationships, wellness, sporting ability, and health. PGT is increasingly accessible online, including in emerging markets such as Australia. Little is known about what consumers expect from these tests and whether their reflections on testing resonate with bioethics concepts such as autonomy.

**Methods:** We report findings from focus groups and semi-structured interviews that explored attitudes to and experiences of PGT. Focus group participants had little experience with PGT, while interview participants had undergone testing. Recordings were transcribed and analyzed using thematic analysis. Findings were critically interpreted with reference to bioethics scholarship on autonomy.

**Results:** Fifty-six members of the public participated in seven focus groups, and 40 individuals were interviewed separately. Both groups valued the choice of PGT, and believed that it could motivate relevant actions. Focus group themes centered on the perceived value of choices, knowledge enabling action and knowledge about the self. Interview themes suggest that participants reflexively engage with their PGT information to make meaning, and that some appreciate its shortcomings. Critical interpretation of findings shows that while consumers of PGT are able to exercise a degree of autonomy in choosing, they may not be able to achieve a substantive conceptualization of autonomy, one that promotes alignment with higher-order desires.

**Conclusions**: PGT consumers can critically reason about testing. However, they may uncritically accept test results, may not appreciate drawbacks of increased choice, or may overestimate the potential for information to motivate behavioral change. While consumers appear to be capable of substantive autonomy, they do so without ongoing support from companies. PGT companies promote a problematic (“default”) account of autonomy, reliant on empowerment rhetoric. This leaves consumers vulnerable to making decisions inconsistent with their higher-order desires. As PGT expands, claims about its power and value need to be carefully drawn.

## Introduction

Personal genomic testing (PGT) is marketed to consumers to promote access to and exploration of genomic information outside of the traditional clinical setting, with or without the assistance of a health professional. People usually purchase these tests online with the intent to explore information about their health, relationships, reproductive risks, ancestry, or wellness (Box 1). PGT originally emerged in the United States, but markets are now well established internationally (Borry and Howard 2008; Borry, Cornel, and Howard 2010; Covolo et al. 2015; Oliveri et al. 2015; Allyse et al. 2018; Howard and Borry 2013). More recent developments include the emergence of online portals where consumers can upload their raw data from non-health testing for re-interpretation, generating health-related data; this is known as third party interpretation, or TPI (Badalato, Kalokairinou, and Borry 2017; Wang et al. 2018; Metcalfe et al. 2019; Nelson, Bowen, and Fullerton 2019).

Australia is also experiencing a growth in PGT, with testing becoming increasingly popular and more easily accessible (Roberts and Middleton 2017). This is partly due to the current regulatory context, where only local companies are required to meet relevant laboratory and test accreditation requirements (Therapeutic Goods Administration 2017). The relevant Australian body, the Therapeutic Goods Administration (TGA), has no jurisdictional control over companies that operate from other countries (Nicol and Hagger 2013; Savard 2013). Consumers are therefore able to import PGT kits and return their DNA sample to the off-shore company for processing and reporting results. There is also ambiguity over whether PGT kits are a “medical device” or an “in vitro diagnostic testing device” under relevant TGA rules, and therefore it is unclear which regulations would apply (Nicol and Hagger 2013; Savard 2013).

PGT might be said to represent a democratization of genomic information, such that an email address, credit card, and postal address are all that is needed to explore our “genomic self” (Nelson 2016). Yet debate continues over ethical and scientific aspects of PGT, such as consent to testing (Bunnik, Janssens, and Schermer 2014); advertising messaging (Schaper and Schicktanz 2018); privacy issues such as the storage, sharing, and future use of consumer data (Laestadius, Rich, and Auer 2017); the utility of test results (Beckman 2004; Bunnik, Janssens, and Schermer 2015; Covolo et al. 2015; Turrini and Prainsack 2016); and the potential for harm (Roberts and Ostergren 2013; Nordgren 2014; Vayena 2015). These debates also take place against a broader background of debate over the readiness of genomics for wider implementation (Burke 2018) and whether genomic information has a substantial positive effect on patient or consumer actions (e.g., Hollands et al. 2016; Stewart et al. 2018).

The concept of autonomy is one that underlies many of these issues. Questions around autonomy have been prevalent in genetic medicine and research, and are now also arising in PGT. For example, aspects of PGT such as the consent process, maintaining control over data, or the utility of results will have implications for whether it can be said to promote or detract from autonomy. There are also more general debates occurring in bioethics regarding how autonomy should be construed, including how a “substantive” account of autonomy can be defended and used in bioethics debates (Dive and Newson 2018). And while they are beyond the scope of this paper to discuss in-depth, it is important to note that autonomy is also relevant to wider ethical and political debates over concepts such as choice and individualism and how they manifest in neoliberal societies like Australia.

Yet while there has been significant quantitative research undertaken with PGT consumers (Savard et al. 2014; Critchley et al. 2015; Goldsmith et al. 2012; Covolo et al. 2015; Savard et al. 2019), there has been less qualitative research; especially with consumers who have actually undergone PGT (Roberts and Ostergren 2013). Additionally, a significant majority of existing research has been undertaken in the United States (Covolo et al. 2015). There has also been little bioethical reflection on how the concept of autonomy manifests in debates on PGT, including the familial and relational implications of these tests. Empirical bioethics research contributes to this discussion by bringing together empirical and normative analysis to allow an opportunity for in-depth critical engagement with consumer expectations and experiences of PGT.

This paper presents and critically reflects on a subset of findings from a larger, mixed-methods project, Genioz (Genomics: National Insights of Australians), described further below, that examined public and consumer expectations and experiences of PGT (Metcalfe et al. 2018). Focus groups explored public awareness and knowledge, while semi-structured interviews explored consumer experiences. The analysis presented here explores participants’ ideas about the choices PGT and its results might enable, and how these findings articulate with bioethical conceptions of autonomy. Exploring these issues contributes to discussions on whether consumers can exercise a substantive conception of autonomy when they pursue PGT.

Box 1 **Types of Personal Genome Tests (PGTs)**

The types of PGTs available for purchase

  - Ancestry* [A]
  - Carrier testing* (including reproductive carrier testing/screening)
  - Drug response / pharmacogenetics / pharmacogenomics
  - Ethnicity-based ancestry testing
  - Food intolerances
  - Health testing, including health-related risk predictions* (e.g., Alzheimer’s disease, blood conditions, heart conditions, other inherited/ genetic conditions) [H]
  - Non-invasive prenatal testing Non-invasive prenatal testing
  - Nutrition and/or wellness (e.g. caffeine metabolism, coeliac disease, oxidative stress, methylation – folate and co-factors, factors affecting weight) [N/W]
  - Paternity
  - Skincare
  - Sporting ability / sporting aptitude / fitness in adults or children [F]

An asterisk (*) indicates the most popular types of genomic tests pursued in Australia (Savard et al. 2019).

[Square brackets] indicate how a test type is abbreviated when referring to qualitative interview participants.

## Methods

### Study design and rationale

The Genioz study aimed to explore the perceptions and use of personal genomics in Australia and to identify gaps in critical thinking that may undermine the informed uses of this technology by consumers (Metcalfe et al. 2018). The study used a staged, mixed-methods approach to obtain a range of qualitative and quantitative data, drawn from focus groups, a national survey, semi-structured interviews and deliberative public workshops. Study components built upon one other. For example, the focus group data informed survey design, and key themes from the survey and interview data determined the topics for the deliberative workshops. There was also an over-arching bioethics analysis across all study components.

This paper presents, analyzes, and critically reflects on two sets of data, drawn from: (i) focus groups involving members of the Australian public (only one of whom had previously had PGT); and (ii) semi-structured interviews with participants who had undergone a form of PGT. These qualitative data have been chosen for analysis as they provide key perspectives and experiences for ethical reflection.

Research ethics approval for both the focus group study and the semi-structured interviews was provided by The University of Melbourne Human Research Ethics Committee (ID 1543685).

### Data collection

Recruitment for focus groups used a variety of approaches, including distributing hard copy posters, dissemination on email lists and advertising on websites and social media (Metcalfe et al. 2018). All focus groups were facilitated by the study chief investigator (SM), in the presence of two note takers. The focus group schedule followed Krueger’s format (1994), which involves starting with an open question to engage all participants. This was followed by a short, 5-minute neutrally framed presentation that introduced the concept of PGT and showed exemplar website home pages for PGT companies which offered ancestry, health, fitness, and lifestyle tests. The focus group interview schedule built on the ideas and concepts from the initial presentation (Metcalfe et al. 2018). Focus groups ran for an average of 1.5 hours, were audio recorded and transcribed.

Interview participants were selected from respondents to the survey that formed the second stage of the overarching study (Savard et al. 2019) who had indicated a willingness to be interviewed. Interviews were conducted by telephone. The interview schedule (See Supplementary File 1) explored participants’ experiences with PGT, including their views on their decision to have testing, the process itself (including test-related information they were given or sought out), how they felt when receiving their results and what their broad views and attitudes were – both about their own test but also the wider enterprise of PGT. Interview duration ranged from 40 to 90 minutes. All were audio recorded and transcribed. Interviews were included in the analysis presented in this paper if the participant: (i) had undergone PGT (including wellness testing, ancestry testing or a combination); (ii) was a “non-specialist” member of the public (e.g., not a laboratory scientist); and (iii) lived in Australia.

### Analysis

Analysis of focus group data was conducted independently by JS and CH using thematic analysis, as described by Braun and Clarke (2013). Themes were derived inductively (“bottom up”) and compared within and between focus groups using a constant comparative approach to generate descriptive codes. Identified codes were discussed further with AN and SM to develop an overall coding framework. Subsequent readings of the focus group data were completed with reference to and guided by the coding framework constructed.

Findings from the focus group analysis also informed the analysis of semi-structured interviews. Focus group themes were adapted by two members of the research team (JS and AN) to develop a deductive (“top down”) coding framework with pre-set codes, which was also informed by debates on autonomy in bioethics. Inductive codes were also noted when they were present and distinct from previously identified ideas. Transcripts were again analyzed with thematic analysis. Coding continued until thematic saturation and consensus between researchers was reached.

Methods of theoretical analysis in bioethics were utilized to critically engage with relevant arguments and literature on autonomy. The outcome of this synthesis was then critically compared with the results of the thematic analysis. This analysis is presented in the Discussion below.

## Results

Seven focus groups involving 56 participants were held in the cities of Melbourne (denoted “M” below) and Sydney (“S”) from July to September 2015. Forty (40) interviews, held between May 2016 and February 2018, met the criteria for inclusion in this analysis. Demographic information regarding participants in the focus groups and interviews is provided in Table 1.

**Table 1.** Demographic information for focus group participants and interviews analyzed.

Demographics	Focus group (n = 56)	Interviews (n = 40)
Age		
18–24	16	0
25–49	14	15
50–80	26	25
Gender		
Female	34	27
Male	22	13
Education		
University Educated (either completed or currently studying)	42	26
Types of Personal Genomic Testing Undertaken*		
Health testing (carrier, serious, non-preventable conditions, serious preventable conditions and pharmacogenomic testing)	1^#^	19
Ancestry (family history tracing and paternity)	1^#^	36
Nutrition and/or wellness testing	1^#^	16
Physical trait and/or fitness testing	1^#^	7

All participants are labeled in-text according to their participant number as it was recorded for the focus groups and interviews (e.g., FGS-X); interview participant labels in text include the type(s) of testing they experienced.

*Many interview participants had experience with more than one test type; therefore, the overall total number of tests taken is greater than the number of interviews analyzed.

^#^This is the same participant.

Focus group and interview participants were collectively interested in a range of PGT, such as health, nutrition/wellness testing, or ancestry. They also indicated similar expectations of what they believed PGT could offer, how they believe they might act upon their results, and what they could do with them. These anticipated and actual experiences are ones where the concepts of choice and knowledge are valorized. Participants from focus groups and interviews also presupposed that actions can (and in many cases will) be taken based upon the information they can obtain from their PGT.

Results from the focus groups and interviews are presented separately, to illustrate the different emphases and attitudes of those with less experience and more experience of PGT. These findings are used in the Discussion to reflect on whether and how PGT might enable consumers to act autonomously.

### Focus groups

Three themes are relevant for this analysis: (i) valuing choice and options; (ii) knowledge enabling action and (iii) knowledge of the self.

#### Valuing the choices and options PGT offers, but with limits

As focus group participants had little experience of testing, many of their comments were anticipating the values of PGT. They framed their ideas about choice to reflect on both the option to have testing, and the options that come from testing. The option to choose to have a test was seen positively:

That’s what I was thinking, choice, choice is one of the good things to come out of it. – FG2M-13 (female)

But to have the choice to know I think is vitally important. – FG4M-35 (female)

Regarding options from testing, participants talked about how they expected that information disclosed from PGT would make certain choices possible. However, participants were also aware that the information obtained in PGT can be perceived in different ways. They saw that it could be (mis)interpreted as constraints imposed by one’s DNA, or that it could be viewed as identifying possibilities:

… most people don’t see [their test results] as a window of possibilities, they see it as a set of limitations, or a set of givens, or a set of restrictions and stuff like that. – FG1M-8 (male)

#### Knowledge from PGT enabling action

Participants did not discuss the (mere) choice to have PGT in isolation. They also connected it to, and valued, the knowledge that PGT might lead to. For example, talk often centered on the value of test results for providing information about one’s future health susceptibilities:

If there’s a disease that I would have a propensity to have and I could change my lifestyle, that would be a benefit. – FG3M-28 (male)

Some participants also articulated that once someone “knows” about certain futures that could be likely (at least according to one’s PGT results), then one could take action:

Absolutely, if I knew I had a predisposition to melanoma, I wouldn’t be going out in the sun. It would change how I, how I do stuff in my life. – FG1M-9 (female)

#### Translating genomic information to genomic knowledge about the self

Although having a choice to access PGT was valued for the actions it might enable, focus group participants were also aware that this might not always be the case. Focus group participants contemplated the consequences that might result when a person tries to make sense of this information. For example, making the results relevant to one's own life and thinking about how gaining this knowledge could lead to uncertainty and cause anxiety:

… it’s I suppose differentiating … between *could* and *will*. If you know you will [get] breast cancer, obviously you will take more serious measures, but if you … get a report that you could get breast cancer, then you spend the next X number of years with sleepless nights thinking: “oh am I going to get breast cancer or not, is today the day?” So, I think that sort of uncertainty … is a little bit of a drawback of answers. – FG1M-7 (male), emphasis added.

Well, I can see the point that, you know, it’s quite hard for me and I think it’s quite hard for others to actually handle that information. Because, if you didn’t have the information, you’d, you’d just go on assuming you didn’t have a risk. If you’re given some information, what actually are you going to obtain, um, as I say, if somebody says to you, you’ve got a 60% chance of having a heart attack, would you, would you avoid exercise? But then if you get another disease because you would have avoided exercise. …[E]ven if it was a yes/no, open/shut – “will I definitely get Alzheimer’s or not?,” well I don’t know, you’ve got a percentage [chance] of getting it…, so does that help people or hinder them, that’s the ethical dilemma. Are you helping people or hindering them by making them, sort of worried-well people. – FG6S-45 (male)

Participants also discussed how this knowledge might influence their lives, including how they would feel about making changes:

Yeah, I don’t like knowing like what’s going to happen, because you change the way you live, because it’s good to know some stuff, but if you want to know everything, you would just change your own life, based around results. – FG1M-5 (female)

While participants in the focus groups had little experience with PGT, their expectations around genomic information either enabling choices or enabling actions illustrates the ways in which genomic information is imbued with value. This narrative is comparable to the ways in which interview participants with experience of PGT talked about their journey of undergoing testing.

### Interviews

Within the 40 interviews analyzed for this paper, many participants discussed how their PGT formed part of a personal investigation or search. For instance, participants who sought ancestry or family history PGT wanted to extend their family history searches or to confirm their current genealogical research, or to (potentially) identify parents in cases of adoption. Similarly, participants who underwent a health, fitness, or wellness test often did so because of a previous “odyssey” to find a diagnosis or cause for their condition; although some also obtained this information via TPI following ancestry testing. For most participants, PGT (whatever the motivation for testing) is incorporated into an ongoing personal exploration, one that continues after results are received. As a result, the motivations and expectations that prompt a person to pursue PGT will influence the ways in which they interpret the “answers” they obtain.

Three themes were developed from interview data: (i) Getting (incomplete) information from PGT, leading to more exploration; (ii) Expecting and making “meaning” from genomic information; and (iii) reflecting on the limitations of the PGT process.

#### Getting (incomplete) information from PGT, leading to more exploration

PGT provided some participants with information they both expected and wanted. However, the ways in which the information was understood, and the role it subsequently played in participants’ lives, varied. For example, some participants discussed how the information they received was not readily comprehensible as received, or was even overwhelming, and required more work to understand:

… so with the DNA test I just need to put more time aside, and then yeah, specific on those tests and try and work out what I- you know, yeah basically go on the Internet and interpret, and see if I can work out what the hell it all means. – Male #1900, N/W

I even sit down and draw diagrams sometimes, I have to [laughs] just to make sense of it. – Female #1965, A

Other participants reported feeling motivated by this multifaceted information. They found their test results to be a useful addition to existing information they had about themselves, or (despite some ongoing lack of clarity) they used it to open new avenues of exploration:

…what did come back is different to what I thought, … well say 50% of it is different to what I thought… but in thinking about it I can … see some logic but there is one aspect of it that I, you know, has got me completely floored, I don’t know what, what it means, so there’s a whole new mystery for me to uncover – Male #2228, A

Likewise, other participants received results that did not meet their expectations, but they could rationalize this:

Because I know that, you know, genetics aren’t your outcome, but it does give you indications where there might be weak spots that you could work on and try and prevent bigger problems down the road. – Female #2376, H & N/W

These adaptive approaches to genomic information demonstrate how consumers can manage and understand the explanatory limits of PGT information. Particularly, interview participants appreciated that a PGT is not a final step, but that it can be the start of a longer engagement with one’s genomic information, such as occurred with participants who had nutrition testing – they continue to interpret and use their test results over time.

#### Expecting and making “meaning” from genomic information

Some participants still expected PGT to provide them with an “answer.” They wanted this information to guide their actions, to define their personal narratives, to tell them something *meaningful*. Within the interviews, participants undertook the process of meaning making from their genomic information by translating the information into actions (when possible) and by incorporating the information into their personal narrative.

For most participants who underwent testing for health, nutrition or wellness, testing was perceived as being very valuable, because it enabled them to progress their diagnostic journey. They perceived their PGT results provided a “label” that only came about because of the test:

…from a health perspective, it gave me answers. As I said, I was desperate, and I was despairing, so it was a relief. It was a massive relief. Um, and I think it was just really empowering, too, because there was something I could do about it. It was wonderful, it was incredibly validating! – Female #2380, H, A & F

In these circumstances, the participants felt the test results validated their lived experiences regarding their health. As a result, information from PGT assumed a confirmatory and explanatory status:

It’s certainly given me a lot of explanation to things that I’ve done … and the things that I prefer to do and things I’m better at, after I’ve read all the medical side of reports I go: “oh whoa, that sort of makes sense!,” a bit like reading an astrology thing but this is actually science that seems to connect quite close to things that have happened in my life. … [It’s] provided a bit more clarity and understanding… – Male #801, H, N/W, A

I would say, it’s not been easy, but it hasn’t been difficult. It’s just um, I’m 60 years of age, so a lot of bad habits have taken place and you know, and all of us have things so to speak, you know, some people develop diabetes or high blood pressure, etc. etc. But knowing where mine comes from, and what I could do um, to not to develop into full blown um, diabetic and um, kidney problems. So, understanding what is happening and working with it. – Female #2376, H & N/W

However, this validation or value was not experienced by all participants:

I thought that it would be more of a “You’ve come up positive for this SNP [single nucleotide polymorphism], therefore your answer needs to be: take this vitamin.” Um, and it hasn’t been like that, it hasn’t been that way at all. – Female #2370, H, N/W, & A

Participants who specifically sought ancestry and family history testing had a similar response. Genomic information enabled them to explore the possibility of genetic connections to particular individuals. This was important for participants who were unable to find these answers through traditional means, such as historical or birth records. To them, DNA is a tool that has “power” – to provide information to investigate potential genetic connections. However, it also impacts upon how these connections are understood. As one participant talked about their newly found genetic relatives:

Yes, yeah and also discover you know what had happened to her life after she had split up with my great grandfather, she went on to have three other children and I also met descendants of those children, we did a DNA test to confirm our relationship um and we’ve been quite close ever since, it’s amazing how, you can show a connection on a family tree, but it’s amazing when you have the DNA result which shows you’re a match, um amazing how that makes you feel towards the other person, you suddenly feel right, it’s not just on paper, it’s, it’s a blood relationship and that it feels a lot stronger, and people, people respond to that I find. – Male #810, H, F & A

This value of genetic information for influencing relationships was also applicable to how one related to themselves:

…I think it’s very interesting in, in some terms it helps kind of re-define how I think of myself, um, in terms of my genetic make-up. – Female #190, A

When the experience was more complex, participants reflected on its perceived value despite the difficulty they faced. One participant reflected on their self-perception changing as a result of testing showing unexpected ancestry:

…that was a bit difficult because I was just not raised with that experience [i.e. that ancestry]… At least I know. And this is the point. It’s important for people to know. And has it helped? It’s helped me to know the truth, and that’s the key to all of it, isn’t it? – Female #2126, H, N/W & A

#### Reflecting on the limitations of the PGT process

Most interview participants approached testing with both excitement and caution. Their caution was articulated when they spoke about the limits of what they believed the information might reveal about themselves or how they might be able to act upon their results. For example, some participants appreciated the shortcomings of the available evidence and how it is interpreted in the reports generated by testing companies:

…there were interesting things like “you are more inclined to get this kind of cancer but also on your genome you also have a great level of protection against this.” So it was like, “wow!” Here we go again, we’re in the hammock and we’re standing up. – Female #2126, H, N/W & A

While many participants approached testing with an expectation that their results could enable action, after receiving results, some were disappointed when there was nothing in their results that they could act upon:

… I felt it really was a bit of a waste of money, not having anything from them personally to act on… – Female #2067, A & F

Look it’s not been a positive experience because it hasn’t really enlightened me in any way at all… it hasn’t really given me any information on that side of the family and that’s really what I was hoping for. … I wished it did, I was really wanting it to, but it really didn’t give me anything. Nothing tangible… – Female #2378, A

Additionally, some participants were concerned about whether the information they received might fail to enable them to make choices relative to, and reflective of, personal preferences regarding their health and life:

Um, yes, you could put it as a concern that “Will this give me enough information to be able, for me, to be able to get what I want for my health benefit?” – Female #2376, H & N/W

Unexpected information was also canvased when talking about ancestry and family history testing – where uncovering events such as misattributed-paternity is possible:

…you get people who, [will] find out your parent isn’t your parent, you get a bit of a shock… I had the potential for that shock although I was fairly confident about who my parents were, … there’s a fair chance that I wasn’t going to get a surprise about my father, but some people do and that’s a risk when you do this sort of test, you might find out a few a few family secrets. – Male #806, H, A & F

It is important to note that many (but not all) interview participants who sought testing were aware of wider implications– such as the potential for third party access to their data. Some participants recognized that their sample would be retained for future analysis, or that their results could become part of the company’s genetic biobank. Having weighed the perceived risks and benefits, they were willing to continue with testing. Other participants, however, appeared to come to realizations about issues such as data privacy during the interview itself. They talked about their concerns around this issue, and identified conditions necessary to enable them to feel comfortable with their decision to undergo testing:

Ah, yes, um, […] I did do a test with [company A] as well because they were doing research into the origins of humankind and um I was quite happy to participate in that, um I would just be concerned with [company B] being a big commercial company doing it for money and um yeah, I didn’t know where it could go to. For [company A] I mean I guess I put some trust in them being a not-for-profit organization and having altruistic aims in terms of their research so, so I tend, I don’t have a principle of not participating in research, you know I’d be happy to do that if it was explained to me what that was about. – Male #810, H, A & F

…up until now it hasn’t really you know, “am I now in a DNA database that the police can access…” that has only just occurred to me right now. I know there’s a lot of legislation, well, I’m assuming there’s a lot of legislation in place …I would assume that it …[privacy legislation] covers information about people’s DNA as well, I could be wrong but I’m relying on it (laughs) so that’s food for thought, I hadn’t actually, it hadn’t occurred to me before, okay, no more spitting in tubes without asking more questions. – Female #1241, A

Overall, interview participants raised some similar issues that prospective consumers identified in the focus groups, in particular the difficulty with seeking and receiving PGT information that may be incomplete. Interview participants understood the information they received as being part of an evolving story – one where they had to “do work” to make sense of their results. Many (but not all) were also mindful that some information could have a negative effect on not only their life, but also the lives of their family members, suggesting a critical awareness when seeking and deciding to undergo testing:

…my basic attitude is that “the test changes nothing.” It’s only telling me what was already happening before I had the test. Um, it’s information, it’s knowledge- knowledge is power. With this knowledge I can move forward – Male #2405, H, N/W & A

## Discussion

This study investigated consumer expectations and experiences of personal genomic testing in Australia. Reflecting broader neoliberal ideology prevalent in countries like Australia, choice and information (the latter often construed as “knowledge”) are valorized. Focus group participants were cautiously positive about both PGT being available and how the information obtained might enable actions, rendering it useful knowledge. However, they also articulated concerns that information from PGT could generate anxiety and that not all information received via PGT may be positive. Interview participants incorporated their PGT test experience into a wider personal narrative, even if the information obtained was not quite what was hoped for. They valued the choice to have a PGT and were motivated to continue interpreting their results over time. They also saw their results as one of many sources of information - valued but usually understood in context. Yet despite this pragmatism, they also imbued PGT results with an explanatory power.

Previous qualitative research on PGT has predominantly been hypothetical or prospective in approach (Goldsmith et al. 2012). Our study has explored in-depth why consumers may seek this testing and how they understand and use it in their daily life. As with a recent study from Schaper, Wohlke, and Schicktanz (2019), it also provides data from participants who are part of the general population, rather than participants from specific settings or groups such as primary health care (Wasson et al. 2013), research (Keller et al. 2010; McBride et al. 2009; Wilde et al. 2010), or early adopters of PGT (Su, Howard, and Borry 2011; McGowan, Fishman, and Lambrix 2010).

As with previous research, this study reports positive consumer views of several forms of PGT. There is enthusiasm for the supposed knowledge that information from PGT can bring (McGowan, Fishman, and Lambrix 2010), including an emphasis on “knowledge” *per se* having power (Fagbemiro and Adebamowo 2014; Wasson et al. 2013). Our results also suggest that PGT may satisfy individual curiosity (Su, Howard, and Borry 2011). Intrinsic to seeking this information is the assumption that information obtained will enable action (McGowan, Fishman, and Lambrix 2010; Su, Howard, and Borry 2011; Wasson et al. 2013). Many participants in this study also appear to have made a decision that aligned with their personal values (Rahm et al. 2012).

The current status quo in the literature is that consumers appear to remain positive about PGT. This is despite ongoing concerns about the utility and scientific validity of the information PGT generates, doubts on whether PGT actually results in relevant actions, complexities in the interrelationship between DNA and personal identity (including identity as an Indigenous person (Watt and Kowal 2019)), and whether PGT promotes individualism at the expense of shared or relational experiences.

Additionally, the extent to which it can be said that consumers are autonomously choosing PGT remains to be addressed. To this end, as well as adding to the body of qualitative research on PGT, our study findings can contribute to ongoing bioethics debates about (consumer or personal) autonomy in relation to PGT.^1^ Personal autonomy, together with related concepts such as personal utility and empowerment, has received some consideration in the PGT literature (Beckman 2004; Juengst, Flatt, and Settersten 2012; Nordstrom et al. 2013; Bunnik 2015; Bunnik, Janssens, and Schermer 2015; Vayena 2015; Loi 2016; Turrini and Prainsack 2016). Two questions are particularly relevant. First, what conceptualization of autonomy might best suit the PGT context? Second, do the findings from our study suggest that consumers can (or do) achieve this?

### What conceptualization of autonomy might best suit the PGT context?

This first question can be addressed by initially looking to how autonomy is being construed in the bioethics literature more generally. Scholars have recently argued that we should move away from the so-called “default account” of autonomy (Dive and Newson 2018). Under the default framing, autonomy focuses more narrowly on the informed consent process and its requirements for information, understanding, and freedom from coercion as the mechanism to achieve an autonomous decision (Hildt 2009; Dive and Newson 2018). This leads to a negative framing of autonomy; one that requires third parties to refrain from certain actions to promote individual autonomy. It also leads to an over-emphasis on the role that information provision plays in autonomy, a point we return to below.

A broader (“substantive”) notion of autonomy, which incorporates elements such as the importance of critical reflection, making authentic decisions, and sensitivity to social context and relationships is increasingly recognized as both important and practicable in bioethics (Dive and Newson 2018). Crucially, this notion of autonomy pays attention not only to the ways in which autonomy is exercised, but the *content* of a decision too. It also looks at whether a person acts in accordance with their higher-order values and desires, rather than merely whether they understood information and were not coerced. As such, this broader understanding of autonomy also allows for autonomy of *persons* (and their higher-order goals and preferences) to be considered, rather than just autonomous *decisions* made by persons.

Both Vayena (2015) and Dive and Newson (2018) posit theorizations of substantive autonomy that draw on Joseph Raz’s *The Morality of Freedom* (1986). Vayena applies her argument to direct-to-consumer testing, while Dive and Newson consider genomic testing more broadly. Key requirements of a Razian theorization of autonomy are that individuals possess appropriate cognitive abilities to form intentions (for both first and higher order goals), have independence to enable un-coerced choices, and are able to choose between an adequate range of critically evaluated options. Thus it is not merely that an individual has options, but that the content of those options has been critically evaluated to be authentically endorsed as meeting higher order desires, such as seeking self-understanding or optimizing health (Juth 2005).

Dive and Newson add, drawing on work by Manson and O'Neill (2007), that autonomy can also only be truly promoted if the communicative context in which a decision is made is appropriate. On this view, communication needs to be an interaction between parties that helps clarify attitudes and promotes understanding. It should not consist solely in the transfer of information. This includes recognizing the assumptions behind statements, beliefs, and desires and not relaying so much information that the consent process is confounded.

A further element of substantive autonomy is attention on relational aspects; namely a recognition of the familial and social connections between individuals and how these shape both who we are and the decisions we make. Relationality is particularly relevant in genomics, because the information obtained is inherently familial. Yet relationality is (somewhat surprisingly) under-considered in discussions of autonomy and genomics (Dove et al. 2017).

Focusing merely on whether a person is competent, informed, and un-influenced (as per the default framing of autonomy) therefore overlooks several relevant aspects of PGT. We will not know whether an individual is fulfilling her higher order preferences when buying a PGT (such as seeking validation of an assumed ancestry, or searching for answers to dietary intolerance), because the default framing does not require us to consider this. The default account’s focus on the decision to test also means autonomy considerations will be limited to the anticipation of the test, not whether autonomy is fulfilled on receipt of results. The underlying rationale for testing is not explored and the social and relational aspects of autonomy are overlooked.

Vayena argues that PGT can be autonomy-enhancing on a Razian conceptualization, especially beyond health tests, because it can provide additional valuable options, increasing the variety of quality choices an individual can make (Vayena 2015). She asserts that an option of PGT can be valuable even if the clinical utility is low, as the personal utility can be valuable. Vayena concedes that this is a defense of PGT in principle, rather than a defense of a specific test offering. In reply, Bunnik rebuts both the validity of a Razian conception of autonomy to PGT *per se* and doubts that PGT could be autonomy-enhancing on this substantive account (Bunnik 2015). She points out that PGT generates “data,” but not necessarily “information” (and even less likely, “knowledge.”) Valuable options, according to Bunnik, need to be those that are “life shaping” – pursuing PGT for enjoyment or to satisfy curiosity would be unlikely to meet this criterion. It should also be recognized that subjective perceptions of utility are not the same as actual utility (whether personal or clinical) (Bunnik 2015), but also that there remains reasonable disagreement on what constitutes utility in PGT (Turrini and Prainsack 2016). Bunnik is concerned that consumers don’t have the chance to critically reflect on this when purchasing a PGT. So, either PGT choices need to offer actual utility; or the conceptualization of autonomy that they allegedly promote needs to be formulated differently.

Drawing this together, to address our first question, a sound conceptualization of autonomy for PGT is not the default one so common in bioethics. Autonomy needs to include the capacity for authentic and critical endorsement of both lower and higher order desires, choosing between a range of quality short- and longer-term options, being free from undue influence, and having a good communicative context (arguably often lacking in PGT (Middleton et al. 2017)) in which to deliberate. Attention should also be paid to the social and relational contexts in which an option is chosen.

This account allows us to see some of the ways in which PGT might not always uphold autonomy. For example, Tutty et al. (2019) show how websites promoting nutrition and wellness PGT market an empowering rhetoric, but do not support this with a good communicative context, including accurate information. Dive and Newson explain why this is problematic:

In order to exercise their autonomy effectively [test recipients] need to reflect critically on the limits and drawbacks of the test, and how obtaining it might align (or not) with their broader goals. (Dive and Newson 2018, 194)

### Can PGT consumers achieve substantive autonomy?

Having shown that a substantive conception of autonomy is appropriate, we now turn to whether our study findings suggest that consumers can (or do) achieve this. Our results suggest that PGT consumers do have the capacity to authentically and critically endorse lower- and higher-order desires. Participants in both focus groups and interviews did not unquestioningly endorse PGT; they appreciated that its utility may be contested and they queried whether PGT would always provide useful information. Focus group participants seemed to value both knowing and not knowing information from PGT, depending on whether such information would be helpful. Interview participants were aware that genetic information had its restrictions and that PGT may uncover things that were not expected. Some interview participants also showed authentic critical reflection on higher order desires when they spoke of how testing informed their personal narratives, such as where their ancestors came from or their perception of why they were experiencing their health symptoms. However, these desires also seemed to affect the framing of their test results, which may have limited their ability to critically reflect upon them.

Participants also did not always recognize that choices should be between a reasonable range of options (per Razian accounts). Participants were instead enthusiastic about choice *per se* and emphasized that testing could lead to more options, but they did not reflect on whether the range of options was reasonable, or whether there is a limit to this. Some also presumed that the information obtained would always engender action or change, an assumption that is not supported by current empirical evidence (Stewart et al. 2018; Hollands et al. 2016). As a result, they reflected less on the potential pitfalls this can bring, or the possibility that information may not effect health behavior changes. Indeed, rather than promoting autonomy, PGT could in fact undermine it.

Regarding the adequacy of the communicative transaction, interview participants (who had participated in such a transaction) were able to see the limits of the test they chose and the potential for other unintended information to arise. However, their reflections on PGT arguably only follow the footprint of the communicative transaction that many companies offer. Participants spoke of finding “the answer,” being “validated,” or “empowered.” The strong empowerment rhetoric in PGT has been commented on extensively in the literature (Liu and Pearson 2008; Hall and Gartner 2009; McGowan and Fishman 2008; Juengst, Flatt, and Settersten 2012; Tutty et al. 2019) and is also reflected in our results. That said, we cannot be sure whether participants have adopted the rhetoric of those providing testing, or whether they believed it already and so were primed to be responsive to it.

Also of note is that participants tended to frame their thinking in an individualistic way, seemingly unaware of the relational aspects to autonomy in genetics and genomics. This is perhaps also an artifact of how PGT is marketed and framed to consumers (Dove et al. 2017). This is particularly interesting because while several interview participants discussed the power vested in DNA to affirm or define relationships, the actual process of testing was viewed as more of an individualistic pursuit.

### Promoting substantive autonomy in PGT

Our data suggest two important aspects for considerations of consumer autonomy in PGT. First, consumers are able to obtain a broad array of autonomy-enhancing factors from testing. This was true even if the test did not seem to give the participant exactly what they desired, or what they had expected from marketing. Second, participants in both focus groups and interviews do have the capacity to critically engage with what PGT promises to do, whether or not they wish to pursue testing. They do not appear to always take company claims at face value; they interrogate these and weigh offerings against each other. They also look to the positive and negative potential implications of testing and (some of) the shortcomings of the tests themselves. However, given that companies appear to rely on and promote a default framing of autonomy (one based largely on one-way information transfer), consumers buying PGT are at risk of not achieving a substantive form of autonomy.

This claim about substantive autonomy and PGT raises the question of whether PGT companies have an obligation to assist their consumers to achieve autonomy on a substantive conception; or whether upholding a “default” autonomy is sufficient. While addressing this question in detail is beyond the scope of this paper, a *prima facie* claim is that companies should not use a strong autonomy or empowerment rhetoric unless they also able to provide support (including a bi-directional communicative transaction) for their consumers to substantively and critically engage with their decisions. This is especially the case when a product can lead to implications for other services, such as health care.

This exploratory study is subject to some limitations. Participants in focus groups were self-selected and had little prior knowledge of PGT. Participants in interviews were purposively selected from those who completed the survey, but survey participants were not purposively sampled. Participants in the study overall were over-represented for higher socio-economic status and higher educational attainment. Therefore, while they offer a rich account of participant views and attitudes and allow a “sensitization” of the bioethical concept of autonomy (Green and Thorogood 2004), the data discussed here are not intended to be generalizable to the opinions of all who have considered or had PGT in Australia. They also provide a snapshot of a particular timepoint in the emergence of PGT. A strength of the Genioz study is the breadth of ages captured by the multiple methods used, as this offers diversity in both ages and ways that participants were able to be involved. For example, participants who may not have been available for a focus group were then captured with the interviews. We were also able to involve people who had a range of experiences with PGT. This range of experiences provides a useful complement to studies with early adopters of PGT.

There are also limitations from using conceptual bioethics reasoning to critically interrogate these data. Other research using methods from sociology, history, political science (and so on) would also provide valuable insights. While we have focused on autonomy – as it is a dominant concept in bioethics scholarship - further analyses are needed to consider how autonomy links with the concept of choice, and the roles of consumerism and individualism in the PGT marketplace.

## Conclusion

Prospective and experienced consumers of PGT in Australia are thoughtfully exploring these tests. The gap between consumer expectations and experiences may not be as wide (or as fraught) as it may have been feared to be. However, while consumers can - and do - successfully navigate PGT, the ways in which it is marketed, sold and accessed risk preventing consumers from achieving substantive autonomy.

The communicative transaction of PGT needs to better promote opportunities for consumers to become substantively autonomous. As PGT expands, rhetorical claims about its power should avoid promoting empty notions of choice and empowerment. Companies should promote a realistic idea of PGT as one element that can facilitate their customers to make authentic choices that align with their values and preferences, and which account for their status as not only individuals but as people who have relational connections too. This means that as the PGT market grows and develops, it will become increasingly important for companies and regulators to actively dampen any “consumerist analogues”^2^ to the therapeutic misconception. PGT companies should also not simply push information to consumers as a one-way transaction. Instead, they should facilitate consumers to critically engage with their choice to have PGT.

Further research is needed to explore PGT in greater depth among those purchasing different test types, such as ancestry or wellness tests. Qualitative research with participants from a more diverse range of socio-economic backgrounds and levels of educational attainment is also required. Such research will assist in determining whether values and engagement with PGT vary between such groups and longitudinally.

Further normative bioethics research should be undertaken to critically explore questions such as whether perceived or objective utility in PGT should matter morally and how bioethical concepts such as autonomy, utility and empowerment relate to and critically inform each other. Bioethicists can also deliberate whether objectively achieving autonomy is important, or whether the subjective perception of having met its substantive components suffices for consumers purchasing PGT.

## Supplementary Material

Supplemental Material

## Data Availability

The data that support the findings of this study will be available from the study lead, Sylvia Metcalfe, upon reasonable request after the study team has completed publications and up until the data storage time limit has been reached.

## Notes

1 As our study involved engaging with consumers and prospective consumers of PGT, our analysis focuses on aspects of autonomy from a consumer perspective. There is, of course, another important element: the behaviors of test companies and the wider transactional context of commercial PGT. Further research is needed to investigate this.

2 We thank one of the reviewers of this paper for offering this phrasing.
