# Supplementary material for: From Expectations to Experiences: Consumer Autonomy and Choice in Personal Genomic Testing
Source: AJOB Empir Bioeth. 2019 Dec 30;11(1):63–76. doi: 10.1080/23294515.2019.1701583 (PMC7048070; doi:10.1080/23294515.2019.1701583)
Supplement: Supplemental Material [file UABR_A_1701583_SM7755.docx]

**Supplementary File 1**

Guide used in semi-structured interviews

| **Before the test** |
| --- |
| - Before we get into the details about the test you purchased, could you tell me why you wanted this particular test? - How did you go about choosing a company to buy your test from? - Who did you talk to about your test before you got it done? - Did you have any concerns about testing? |
| **The testing process** |
| - How long ago did you undergo testing? - How was your test ordered? - What did you think about the cost of the test? - What was it like to have the test kit arrive in the post? - Can you tell me what you remember about the terms/conditions when you registered the test? - After submitting your sample, did you think about what the results might say? |
| **After the test** |
| - When waiting for your results, did you think about the test at all? - Tell me about the day your results were ready? What was it like to get the email/phone call? - Were the results what you expected to get? What was different to what you expected to get? - Which result was the most interesting? Did you find any results useful? Which ones? - What did the information look like? Was it easy to read and understand? - How accurate do you think the results were? - Now that you have this information, what do you plan to do with it or have done with it? - In hindsight, do you think you had enough information provided to you when you were deciding whether or not to be tested? - Have you shared this information with anyone? - If not, why not? - Was there anything unexpected that happened after testing? |
| **Supports and information needs** |
| - If someone was thinking about getting this test, what advice would you give them? - Are there other forms of information about testing you would have liked to access or be able to get – either before, during or after testing? - At the end of the survey, we provided some links to further information, I’m wondering if you looked at any of these? - After testing, did you look elsewhere for information? - How do you feel about these testing services being offered by a commercial company? - Do you feel there is adequate rules and governance around these tests? - If you had an issue with the test – where do you think you might go for help if you had a complaint or wanted to seek guidance about your test results? |
